# Formulation and Evaluation of Omeprazole Tablets for Duodenal Ulcer

**DOI:** 10.4103/0250-474X.73922

**Published:** 2010

**Authors:** A. Choudhury, S. Das, S. Bahadur, S. Saha, A. Roy

**Affiliations:** Department Pharmaceutics, GRY Institute of Pharmacy, Borawan, Khargone - 451 228, India

**Keywords:** Diffusion, enteric-coating, erosion, mucoadhesion, omeprazole, pellet, ulcer

## Abstract

Omeprazole pellets containing mucoadhesive tablets were developed by direct punch method. Three mucoadhesive polymers namely hydroxypropylemethylcellulose K4M, sodium carboxy methylcellulose, carbopol-934P and ethyl cellulose were used for preparation of tablets which intended for prolong action may be due to the attachment with intestinal mucosa for relief from active duodenal ulcer. Mucoadhesive tablets were coated with respective polymer and coated with Eudragit L100 to fabricate enteric coated tablets. The prepared tablets were evaluated for different physical parameters and dissolution study were performed in three dissolution mediums like 0.1N hydrochloric acid for 2h, pH 6.5 and pH 7.8 phosphate buffer solution for 12hr. Sodium carboxymethylcellulose showed above 95% release within 10 h where as carbopol-934P showed slow release about 88% to 92% over a period of 12 h. having excellent mucoadhesive strength but ethyl cellulose containing tablets showed less than 65% release. The release mechanism of all formulation was diffusion controlled confirmed from Higuchi’s plot. Thus, the present study concluded that, carbopol-934P containing mucoadhesive tablets of omeprazole pellets can be used for local action in the ulcer disease as well as for oral controlled release drug delivery.

Omeprazole, a substituted benzimidazole, is a potent inhibitor of gastric acid secretion by selectively interacting and inhibiting the gastric parietal cell proton pump[1]. Omeprazole was widely used in the treatment of active duodenal ulcer, active benign gastric ulcer, gastro-esophageal reflux disease (GERD), erosive esophagitis and other pathological hypersecretory conditions[2]. Omeprazole degrades in water but is readily soluble in alkaline conditions[3]. The stability of omeprazole decreases in acidic medium, when it comes in contact of acidic medium leads a significant degradation of the drug and hence reduced bioavailability. Due to its low bioavailability, short biological half life[4] and hepatic first pass metabolism, various oral formulation of omeprazole such as enteric-coated granules[5,6] and tablets[7,8] have been developed with a subsequent 40% increase in oral bioavailability[9] of omeprazole but have a wide individual variation of plasma concentration in human[5–8]. To overcome this problem, alternative dosage forms such as rectal suppository[10] and buccal adhesive tablets[11] were also developed. But all the dosage form of omeprazole gives only systemic effect, hence, attempts have been made to develop enteric coated mucoadhesive sustained release product which increase residence time due to attachment with the intestinal mucosa for prolong time and may give local effect in duodenal ulcer, reduced drug loss and also reduced dosing frequency.

Omeprazole pellets were a gift sample from Diamond Drugs Pvt. Ltd., Kolkata, India. Eudragit L100 was a gift sample from Zydus Cadila, Rangpo, India. Hydroxypropylmethylcellulose K4M (HPMC-K4M) and sodium carboxymethylcellulose were supplied by Loba Chemie Pvt. Ltd., Mumbai and Visco Industry, Houra, India, respectively. Ethyl cellulose- LR (EC-LR) and Carbopol 934P were supplied by S. D. Fine-Chem. Limited, Mumbai, India.

Drug and polymer interaction study was carried out using Fourier Transform Infrared (FTIR-8400S, Shimadzu Corporation, Japan) and sample scanned between 4000-400 cm^-1^ at a resolution of 2 cm^-1^. The principal IR absorption peaks of omeprazole pallets at 1016, 1205 and 1627 cm^-1^ were obtained in the spectra of the pure drug as well as different drug -polymer complex indicating that no chemical interaction occurred between the omeprazole pallets and the polymer used[3].

Required quantity of omeprazole pellets were mixed with the respective polymer as mentioned in Table 1 for the preparation of formulation F1, F2, F3 and F4. Di-basic calcium phosphate was mixed with the formulations in desired amount and blended for 30 min. Finally talc was mixed with each blending just prior to punching tablets. Two hundred milligram tablets were fabricated as per the formula given in Table 1 using a tablet compression machine (Rimek Mini Press-I, Shakti Engineering). Each formulation was coated with 0.5% solution of respective polymers (hydroxypropylmethylcellulose K4M, sodium carboxymethylcellulose, ethyl cellulose and Carbopol 934P) and the tablets were made enteric-coated tablets by coating with 0.5% solution of Eudragit L100. Coating was done by dipping the tablets into the 0.5% coating solution and immediately drying under hot air flow[12].

**TABLE 1:** COMPOSITION OF MUCOADHESIVE TABLETS OF OMEPRAZOLE PELLETS

Formulation	Ingredient (gm/tablets)						
	Omeprazole pellets	HPMC	NaCMC	EC	Carbopol 934P	Di-basic calcium phosphate	Talc
F1	0.0914	0.0457	--------	-------	-------	0.0609	0.002
F2	0.0914	-------	0.0457	-------	-------	0.0609	0.002
F3	0.0914	-------	-------	0.0457	-------	0.0609	0.002
F4	0.0914	-------	-------	-------	0.0457	0.0609	0.002
				

Thickness and diameter, hardness and friability were determined before and after coating using a slide calipers, Monsanto hardness tester and Roche friabilator, respectively. The swelling index was calculated using the formula[12–14], swelling index= (W2-W1)/W1 where, W1 and W2 is the initial weight and soaked weight, respectively. Drug content in the formulation was calculated by UV spectrophotometric (UV-1700, Shimadzu Corporation, Japan) method based on the measurement of absorbance at 300 nm in pH 6.5 and pH 7.8 phosphate buffer solutions[12,14,15]. Mucoadhesive strength was also determined by modified physical balance, where weight required to detach the tablet from the mucosal surface was taken as the measure of mucoadhesive strength[12,14,16].

The release rates of prepared enteric-coated mucoadhesive tablets of omeprazole were studied using a Veego dissolution test apparatus (USP II) rotating paddle method under sink conditions at 37±0.5° and 50 rpm speed. Tablets were placed in the basket and tested for drug release up to 2 h in 0.1N hydrochloric acid solution and for 12 h in pH 6.5 and 7.8 phosphate buffer solution. Analysis of the drug content in the solution was performed spectrophotometrically[12–15].

The formulation had low tablet weight variation (% deviation < 0.5). Hardness of the tablets was found in the range 6 to 7 kg/cm^2^ and percent weight loss in the friability test was ≤ 0.08 in all the batches. Highest % swelling index of 1.51 was obtained with the formulation containing carbopol 934P. Drug content of the tablets in all the batches was found to be 89.66 to 91.33%. Overall, the prepared tablet batches were of good quality with respect to hardness, friability, weight uniformity and drug content. Enteric coating trials yielded tablets without edge defects or surface imperfections. Detachment force measurement method was used for the determination of mucoadhesive strength of different formulations and it was observed that carbopol 934P offered the highest bioadhesive strength (between 30-31 g) and sodium carboxymethylcellulose (between 20-22 g) provided good bioadhesive property but ethyl cellulose did not give any bioadhesive property. All physical evaluation parameters of the tablets have been shown in Table 2.

**TABLE 2:** PHYSICAL EVALUATION OF COATED AND ENTERIC-COATED MUCOADHESIVE TABLETS

Formu-lation	Average Weight variation (mg)	Average Friability	Average Hardness (kg)	Drug content (%)	% Swelling index	Mucoadhesive strength (gm)	Release rate (%)
F2	199.86±0.3	0.07±0.01	6.6±0.1	89.66	0.56	21±0.5	95
F3	200.11±0.5	0.08±0.01	6.8±0.2	90.21	0.12	02±0.5	67
F4	199.42±0.5	0.07±0.01	6.2±0.2	91.33	1.51	30±0.5	92

The release of omeprazole from the tablets was studied in phosphate buffer at pH 6.5 and 7.8 using the dissolution apparatus USP II. Mucoadhesive tablets containing hydroxypropylmethylcellulose, sodium carboxymethylcellulose, carbopol 934P alone gave slow release over a period of 10, 8 and 10 h, respectively. After comparing all the data of two formulations (hydroxypropylmethylcellulose, carbopol 934P) it was observed that these tablets had similar release pattern. However, tablets containing water insoluble polymer ethylcellulose released the drug to a maximum of 80% over a period of 10 h. Coated as well as enteric-coated tablets were prepared and the release profiles of these tablets were studied in three media, 0.1N HCl, pH 6.5 and 7.8 phosphate buffer solutions. The hydroxypropylmethylcellulose containing formulation was discarded due to inconsistent process variables. Simple coated and enteric-coated tablets were prepared and were found to be acid resistance since percent of omeprazole release in 0.1N HCl medium did not exceed 17% over 2 h (fig. 1). From the release profile data it was found that sodium carboxymethylcellulose containing tablets gave a minimum 95% release over a period of 8 to10 h, whereas carbopol 934P containing tablets released the drug to a maximum 92% over a period of 12 h and ethylcellulose containing tablets released less which was found to be not more than 67% over a period of 12 h. (figs. 2 and 3). On the basis of these drug-release profiles, mechanism of drug release was confirmed by Higuchi’s plots (fig. 4) that showed graphical representation of cumulative percent drug release versus square root of time. The Higuchi’s plots were found to be linear with correlation coefficient values of 0.974, 0.983, 0.983 and 0.974, 0.956, 0.976 for F2, F3 and F4 in pH 6.5 and 7.8, respectively. From the Higuchi’s plots it was concluded that the release of drug was diffusion-controlled in all formulations.

**Fig. 1:**
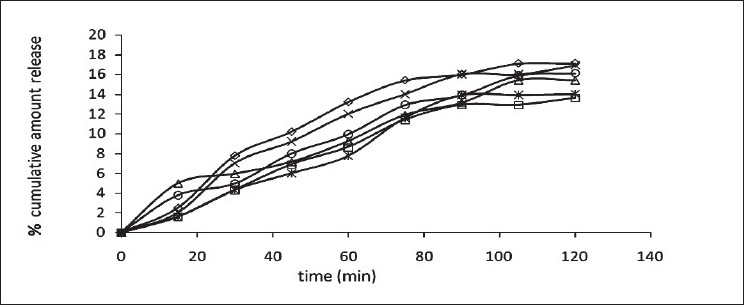
*In vitro* release profile of mucoadhesive tablets in 0.1 N HCl Enteric-coated formulations, F2 (–◊–), F3 (–□–) and F4 (–Δ–) and simple coated formulations, F2 (–×–), F3 (–☆–) and F4 (–○–)

**Fig. 2:**
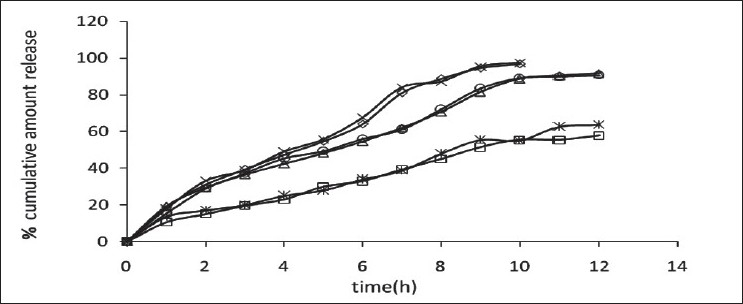
*In vitro* release profile of coated mucoadhesive tablets Release in pH 6.5 phosphate buffer from formulations, F2 (–◊–), F3 (–□–) and F4 (–Δ–), and release in pH 7.8 phosphate buffer from formulations, F2 (–×–), F3 (–☆–) and F4 (–○–)

**Fig. 3:**
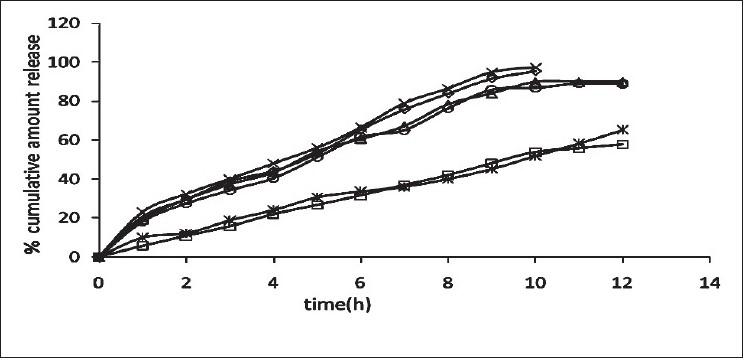
In vitro release profile of enteric-coated mucoadhesive tablets Release in pH 6.5 phosphate buffer from formulations, F2 (–◊–), F3 (–□–) and F4 (–Δ–), and release in pH 7.8 phosphate buffer from formulations, F2 (–×–), F3 (–☆–) and F4 (–○–)

**Fig. 4:**
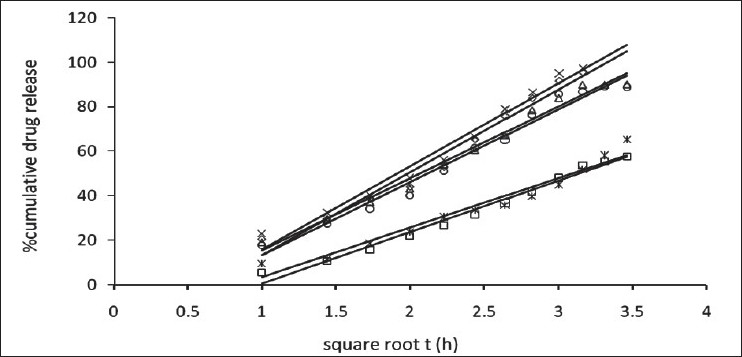
Higuchi’s plot of enteric coated mucoadhesive tablets Release in pH 6.5 phosphate buffer from formulations, F2 (–◊–), F3 (–□–) and F4 (–Δ–), and release in pH 7.8 phosphate buffer from formulations, F2 (–×–), F3 (–☆–) and F4 (–○–)

In this study meaningful results were obtained after performing physical evaluation of mucoadhesive tablets of omeprazole pallets except for drug content since it is likely that some amount of drug might have been released in to the coating solution during coating. It can be concluded that the tablets containing 20 mg omeprazole with carbopol 934P (F4) coated with Eudragit L100 showed good % swelling index i.e. 1.51, satisfactory drug content of 91.33%, promising mucoadhesive strength of about 30 g and convenient controlled drug release in 12 h that is 92%, thus appear to be a potential candidate for the development of mucoadhesive tablet for effective therapeutic use as well as controlled drug delivery.
